# Supplementary material for: Atypical hippocampal excitatory neurons express and govern object memory
Source: Nat Commun. 2025 Feb 12;16:1195. doi: 10.1038/s41467-025-56260-8 (PMC11822006; doi:10.1038/s41467-025-56260-8)
Supplement: Supplementary file 1 — Supplementary Information [file 41467_2025_56260_MOESM1_ESM.pdf]

Title: Atypical hippocampal excitatory neurons express and govern object memory

Supplementary Figures:

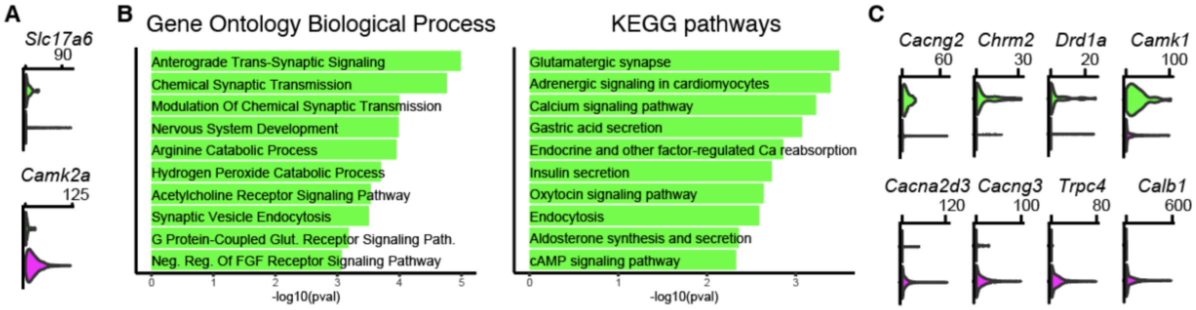

Supplemental Fig. 1. Transcriptomic specializations of ovoid neurons.

(**A**) Expression of excitatory neuron markers *Camk2a* and *Slc17a6*, visualized via violin plots and quantified in counts per million. (**B**) Analysis of biological processes from gene ontology (left) and KEGG pathways (right) for genes enriched in the outlying subtype. (**C**) Example differentially expressed genes involving in calcium signaling.

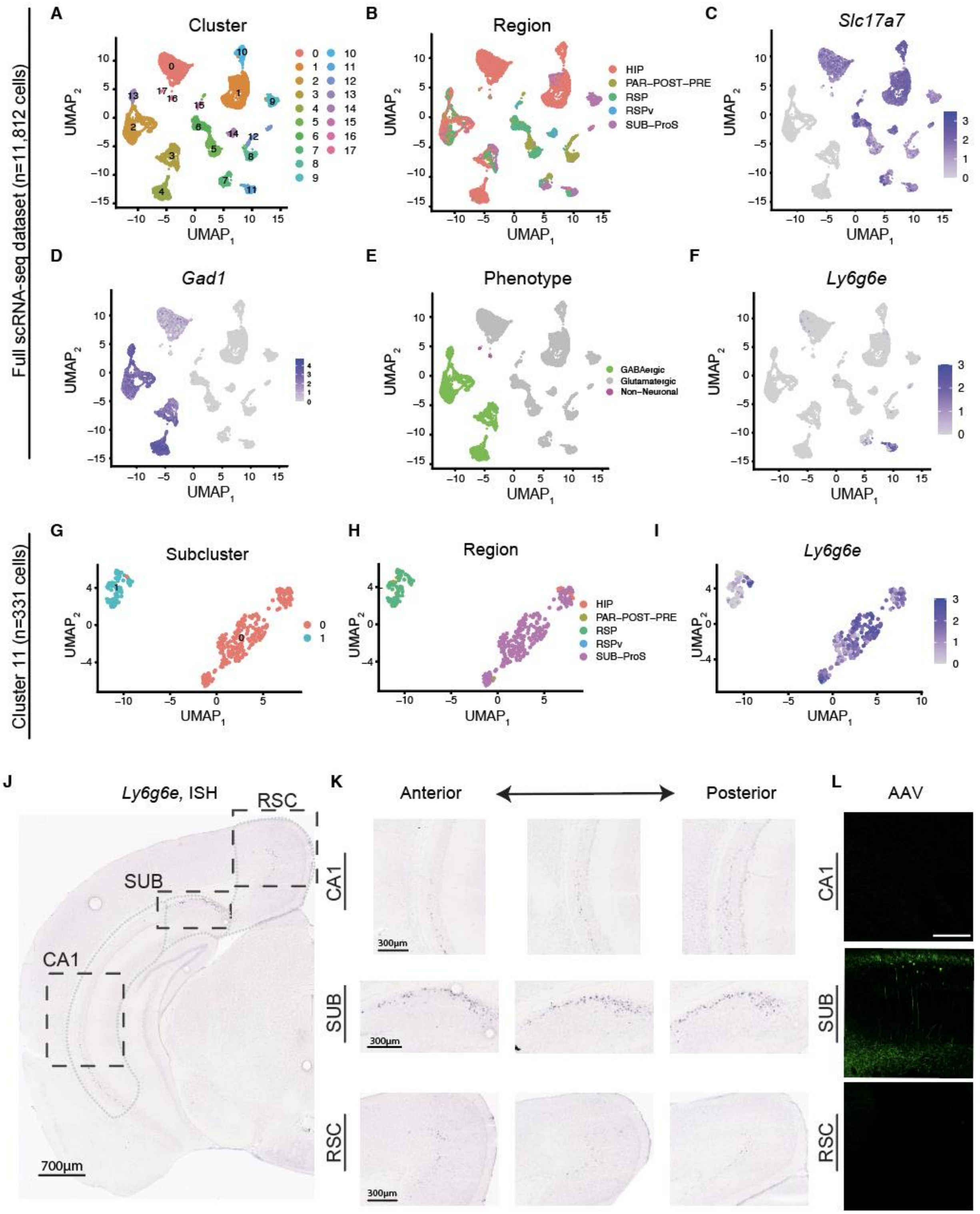

Supplemental Fig. 2. ***Ly6g6e*** **provides selective access to subiculum ovoid neurons.**

(**A**) UMAP embedding and clustering of hippocampal formation and retrosplenial cortex neurons. Data from [74]. (**B**) As in (A), but plotted by region of harvest. HIP: hippocampus, PAR-POST-PRE: parasubiculum, postsubiculum, and presubiculum; RSP: retrosplenial cortex; RSPv: ventral retrosplenial cortex; SUB-ProS: subiculum and prosubiculum. (**C**) Expression of the excitatory neuron marker *Slc17a7*. (**D**) As in C, but for the inhibitory neuron marker *Gad1*. (**E**) Cellular phenotype, as assigned from [74]. (**F**) As in C, but for the ovoid neuron marker *Ly6g6e*. (**G**) Reclustering analysis of *Ly6g6e*-expressing cluster 11 from A-F. Colours denote subcluster identity. (**H**) As in B, but for the reclustering analysis of Cluster 11. (**I**) As in F, but for reclustering analysis of Cluster 11. (**J**). Expression of *Ly6g6e* from *in situ hybridization.* Data from [31]. CA1, subiculum (SUB), and retrosplenial cortex (RSC) regions are shown. (**K**) Expansions of CA1, SUB, and RSC for brain sections across the anterior-posterior axis. (**L**) AAV-mediated Cre-dependent GFP labeling for CA1, SUB, and RSC.

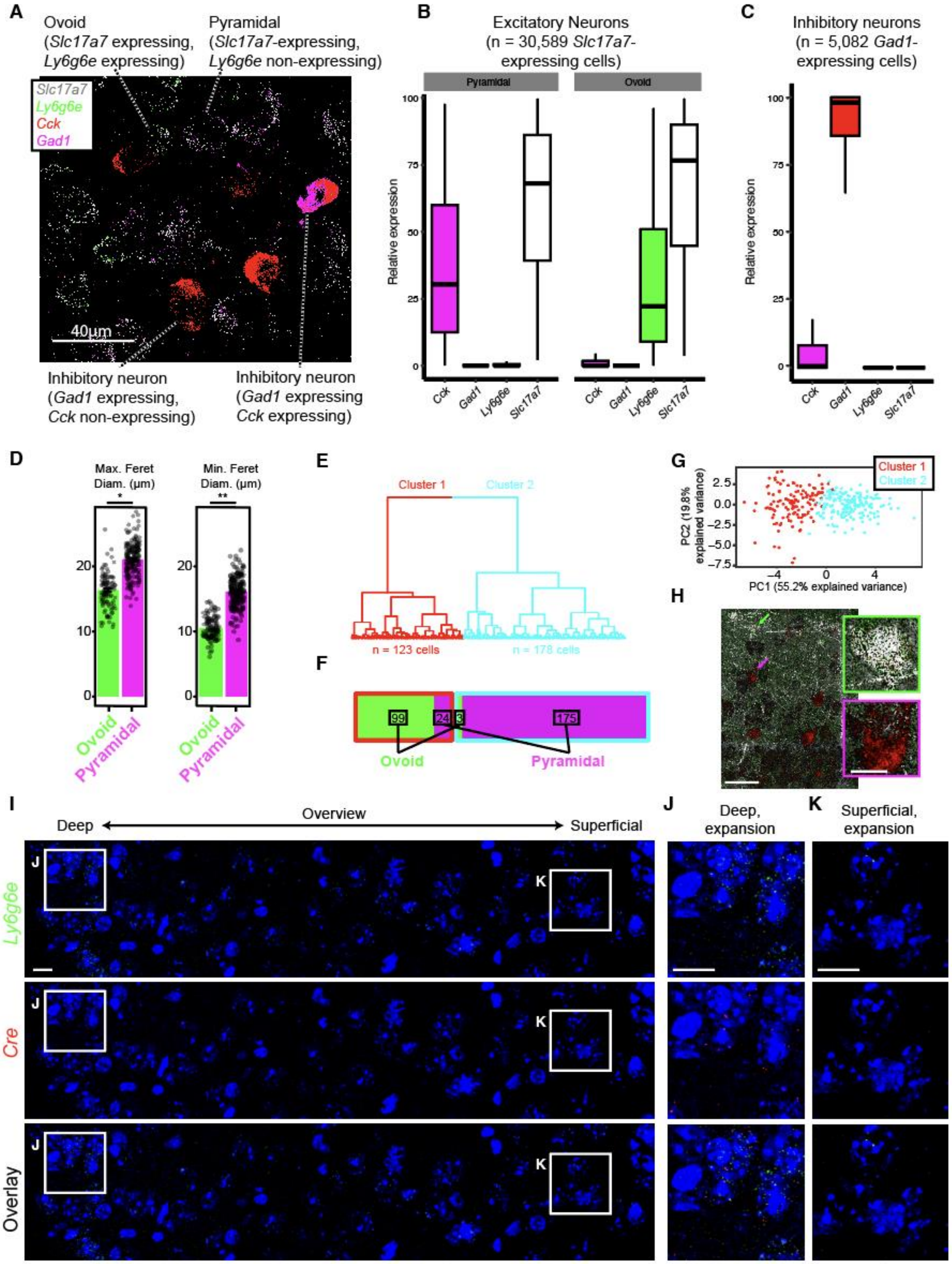
A
Ovoid (Slc17a7 expressing, Ly6g6e expressing)
Pyramidal (Slc17a7-expressing, Ly6g6e non-expressing)
Slc17a7
Ly6g6e
Cck
Gad1
40µm
Inhibitory neuron (Gad1 expressing, Cck non-expressing)
Inhibitory neuron (Gad1 expressing Cck expressing)
B
Excitatory Neurons (n = 30,589 Slc17a7-expressing cells)
Pyramidal
Ovoid
Relative expression
Cck
Gad1
Ly6g6e
Slc17a7
C
Inhibitory neurons (n = 5,082 Gad1-expressing cells)
D
Max. Feret Diam. (µm)
Min. Feret Diam. (µm)
Ovoid
Pyramidal
E
Cluster 1
Cluster 2
n = 123 cells
n = 178 cells
F
99
24
3
175
Ovoid
Pyramidal
G
PC2 (19.8% explained variance)
PC1 (55.2% explained variance)
H
I
Deep
Overview
Superficial
Ly6g6e
Cre
Overlay
J
Deep, expansion
K
Superficial, expansion

Supplemental Fig. 3. mFISH-based readouts of ovoid neuron cell-body geometry, connectivity, and gene expression properties.

(**A**) mFISH image and example cellular phenotyping based upon expression of *Slc17a7*, *Ly6g6e*, *Gad1*, and *Cck*. (**B**) Expression of marker genes in *Slc17a7*-expressing putative excitatory neurons. (**C**) As in B, but for *Gad1*-expressing neurons. (**D**) Summary of cell-body properties for ovoid neurons and pyramidal neurons, as assayed via correlative mFISH-Nissl staining (max feret p = 3.9e-2; min ferret p = 4.6e-3 2 via two-sided paired t-test on within-animal-averaged data). Data are presented as mean values +/- SEM. (**E**) Dendrogram illustrating hierarchy of cell-body organization, colored according to primary bifurcation. (**F**) Proportion of ovoid and pyramidal cells in each cluster, as assayed by mFISH marker gene expression. Numbers denote total cells within each group. (**G**) Principal component analysis of cell bodies, colored according to cluster identity from B. Cluster 1 (*Ly6g6e*-expressing) 116 cells; cluster 2 (*Cck*-expressing): 185 cells. (**H**) *Ly6g6e* (green) and *Cck* (red) labeling from mFISH, in conjunction retrograde labeling for ATN-projecting neurons (white), across the pyramidal cell layer of the subiculum. Example cell bodies are highlighted in expansions, corresponding to *Ly6g6e*-expressing ovoid neurons and *Cck*-expressing pyramidal cells (green and magenta boxes, respectively, denoted by arrows in overview image). Right: Scale bars: 50 µm overview, 10 µm expansion. (**I**) mFISH imaging of *Ly6g6e* (green) and *Cre* (magenta) across the superficial-deep axis in the Ly6g6e-IRES-Cre mouse line. Blue represents DAPI counterstain. Scale bar: 10 µm. (**J**) Expansion of deep subiculum region denoted in I. Scale bar: 10 µm. (**K**) As in J, but for superficial subiculum. For I-K, results are representative of n=3 animal replicates.

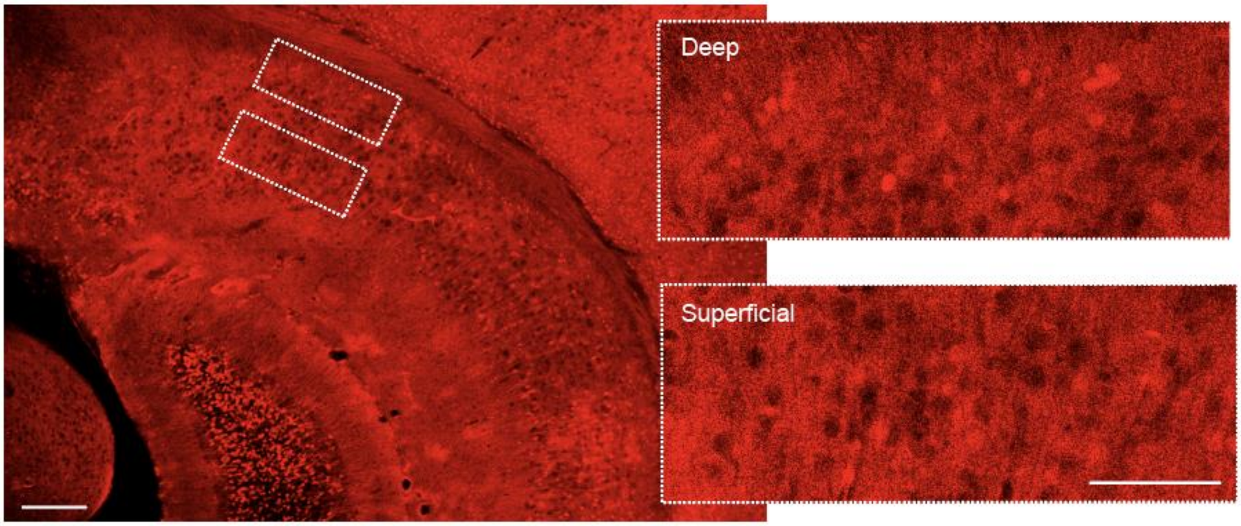

Supplemental Fig. 4. Cre-dependent tdTomato expression following transgenic reporter cross.

Left: overview of tdTomato expression following Ly6g6e-IRES-CRE cross to the Cre-reporting Ai14 tdT reporter strain. Scale bar: 200 µm. Right: expansions of deep subiculum (top) and superficial subiculum (bottom), each illustrating labeled cell bodies and broad tdTomato background. Scale bar: 100 µm.

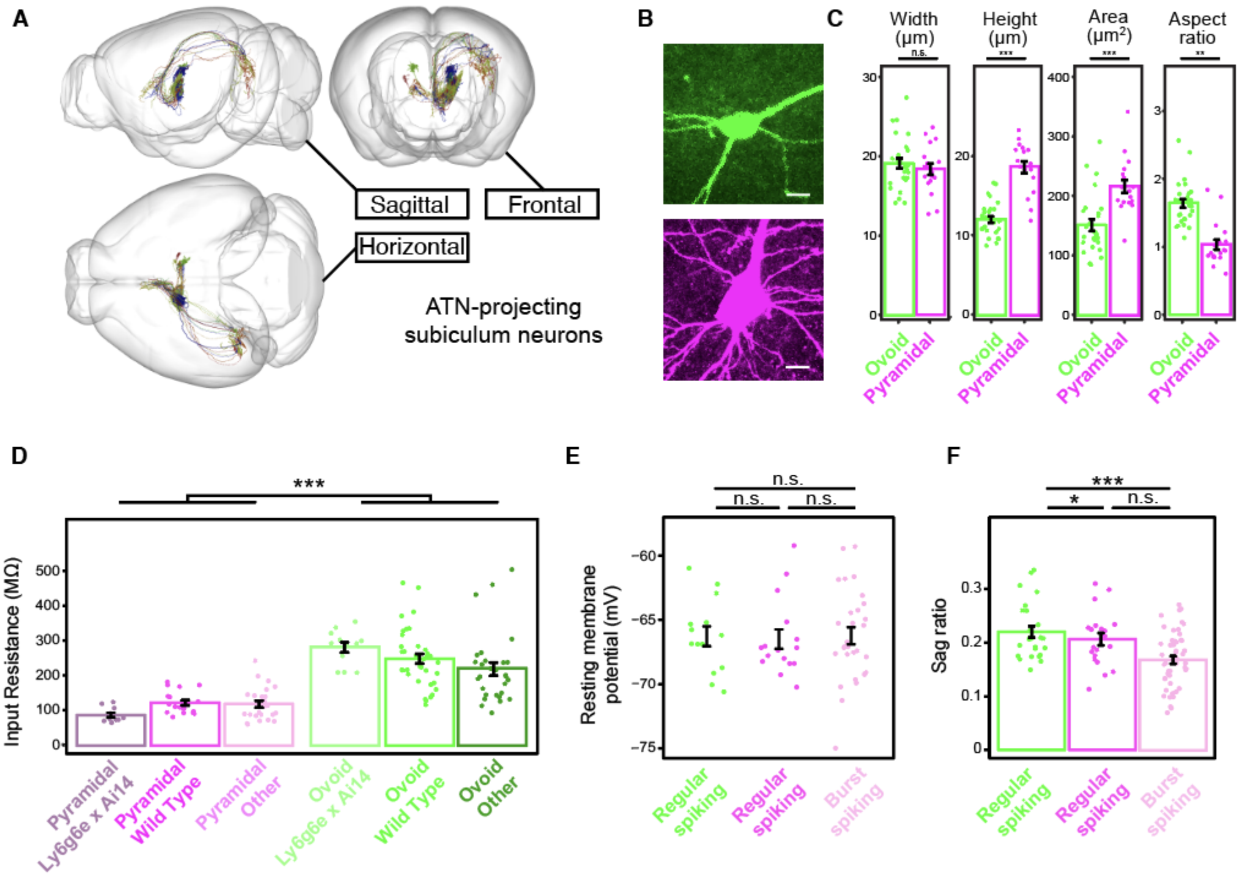

Supplemental Fig. 5. Anatomical and electrophysiological properties of ovoid neurons.

(**A**) Illustration of ATN-projecting subiculum neurons in horizontal (left), sagittal (middle), and frontal (right) planes. (**B**) Example cell bodies for ovoid (green) and pyramidal (magenta) neurons, as obtained from cell reconstructions via whole-cell patch-clamp recordings. Scale bar: 5 µm. (**C**) Summary of cell-body properties for ovoid neurons and pyramidal neurons, as obtained from cell reconstructions via whole-cell patch-clamp recordings (n = 40 ovoid, n =27 pyramidal; width: p = 0.40; height: p = 2.5e-9; area: p = 8.1e-6; aspect ratio: p = 3.8e-3 via two-sided Mann-Whitney U test on within-animal-averaged data). Data are presented as mean values +/- SEM. (**D**) Input resistance of cells recorded *ex vivo*. Pyramidal neurons (first three entries) and ovoid neurons (last three entries) were recorded from double-positive Ai14xLy6g6e mice, wild-type mice, and other mice (i.e. mice from Ai14xLy6g6e breeding that were not double positive; n = 67 total animals, data points represent individual animals; pooled pyramidal vs. ovoid analysis: p<2.2e-16; via Kruskal Wallis test on within-animal-averaged data). Data are presented as mean values +/- SEM. (**E**) Resting membrane potential of cells recorded *ex vivo* (n = 37 animals; ovoid vs. regular spiking pyramidal p = 0.60; ovoid vs. burst spiking pyramidal p = 0.95; regular spiking pyramidal vs. burst spiking pyramidal p = 0.52, via Kruskal Wallis test on within-animal-averaged data). Data are presented as mean values +/- SEM. (**F**) As in E, but for sag ratio (n = 67 animals; ovoid vs. regular spiking pyramidal p = 3.0e-2, ovoid vs. burst spiking pyramidal p = 6.7e-4 regular spiking pyramidal vs. burst spiking pyramidal p = 0.59, via

Kruskal Wallis test on within-animal-averaged data). Data are presented as mean values +/- SEM.

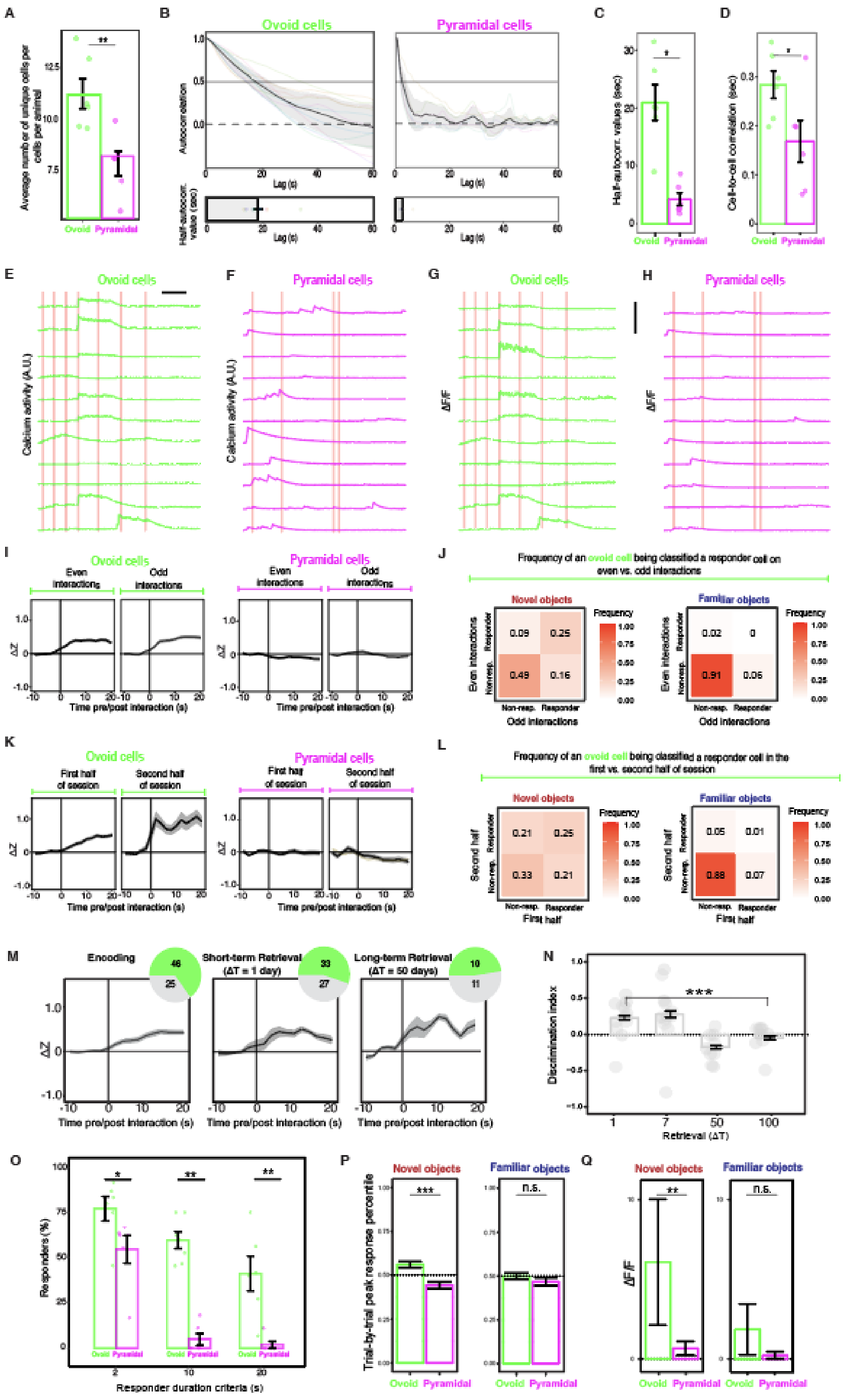

Supplemental Fig. 6. Ovoid neuron activity across behaviors, sessions, and animals.

(**A**) Number of unique cells recorded per animal, for imaging sessions targeted to ovoid neurons (green) and pyramidal neurons (magenta) (n = 6 ovoid, n = 6 pyramidal $p = 0.01$, two-tailed Mann-Whitney U test). Data are presented as mean values +/- SEM. (**B**) Top left: Autocorrelation of cellular activity in ovoid neurons from one animal during a 1-day retrieval session. Individual traces represent individual cells, black line and grey ribbon illustrate mean ± SEM across cells. Bottom left: Barplot summary illustrating half-correlation values, summarised across cells. Right: as at left, but for pyramidal neurons in a representative recording session. (**C**) Summary of half-autocorrelation values across all animals for a representative (1-day retrieval) session (n = 6 ovoid, n = 6 pyramidal; $p = 5.1\text{e-}3$, two-sided Mann-Whitney U test). Data are presented as mean values +/- SEM. (**D**) Summary of cell-to-cell correlation values, averaged across all recording sessions (n = 6 ovoid, n = 6 pyramidal; $p = 0.026$, two-sided Mann-Whitney U test). Data are presented as mean values +/- SEM. (**E**) Example calcium activity (in arbitrary units, "A.U.") of ovoid neurons following novel object interactions. Individual cells are illustrated in colored traces, with novel object interactions illustrated by red vertical lines. Scale bar: 30 seconds. (**F**) As in E, but for pyramidal cells. (**G,H**) As in E,F but for ΔF/F values, with baseline F values identified globally across the duration of the experiment. Scale bar: 10. (**I**) Cellular activity in response to novel object encounters, summarized across all animals, based upon subsetting odd (left) and even (right) interactions with novel objects. Ovoid neurons are depicted at left (green), and pyramidal neurons at right (magenta). (**J**) Summary of responder vs. non-responder cells, as categorized in odd and even interaction subsets. (**K,L**) As in I,J but for interactions occurring in the first and second half of individual behavior sessions. (**M**) Novel-object-induced cellular activity in ovoid neurons, averaged across all cells on a per-animal basis across encoding, 1-day retrieval, and 50-day retrieval (left, middle, and right respectively). Individual lines illustrate individual animals, black line and grey ribbon illustrate mean ± SEM across animals. Inset charts illustrate total number of cells that classify as responders, summarized across all recorded animals. (**N**) Discrimination index as a function of retrieval day, summarized across all imaged animals. Individual points illustrate individual animals (n = 6 ovoid, n = 6 pyramidal; $p = 4.3\text{e-}4$, Kruskal-Wallis Test). Data are presented as mean values +/- SEM. (**O**) Percent of neurons that classify as responders for minimum duration criteria of 2, 10, and 20 seconds. (n = 6 animals in each case; ovoid vs. pyramidal at 2 second minimum duration criteria $p = 3.7\text{e-}2$, 10 second criteria $p = 2.3\text{e-}3$, 20-second criteria $p = 4.8\text{e-}3$, Kruskal-Wallis test). Each dot is an animal and error bars represent standard error. (**P**) Left: peak trial-by-trial responses for all cells following novel object encounters across a 1-day retrieval session, provided in terms of percentiles relative to the full recording session. Right: as at left, but for familiar object encounters (n = 269 pyramidal, n = 273 ovoid; novel: control vs ovoid $p = 7.8\text{e-}6$, ovoid vs 0.5 $p = 6.6\text{e-}4$, control vs 0.5 $p = 2.9\text{e-}3$, familiar: control vs ovoid $p = 0.19$, ovoid vs 0.5 $p = 0.93$, control vs 0.5 $p = 0.065$, two-sided Mann-Whitney U Test). Data are presented as mean values +/- SEM. (**Q**) Left: ΔF/F values following novel object encounters across a 1-day retrieval session, with baseline F values defined as pre-encounter activity. Right: as at left, but for familiar object encounters (n = 33 pyramidal; n = 51 ovoid; novel: control vs ovoid $p = 9.8\text{e-}3$, ovoid vs 0 $p = 8.3\text{e-}6$, control vs 0 $p = 0.48$, familiar: control vs ovoid $p = 0.67$, ovoid vs 0 $p = 0.13$, control vs 0 $p = 0.67$, two-sided Mann-Whitney U test). Data are presented as mean values +/- SEM.

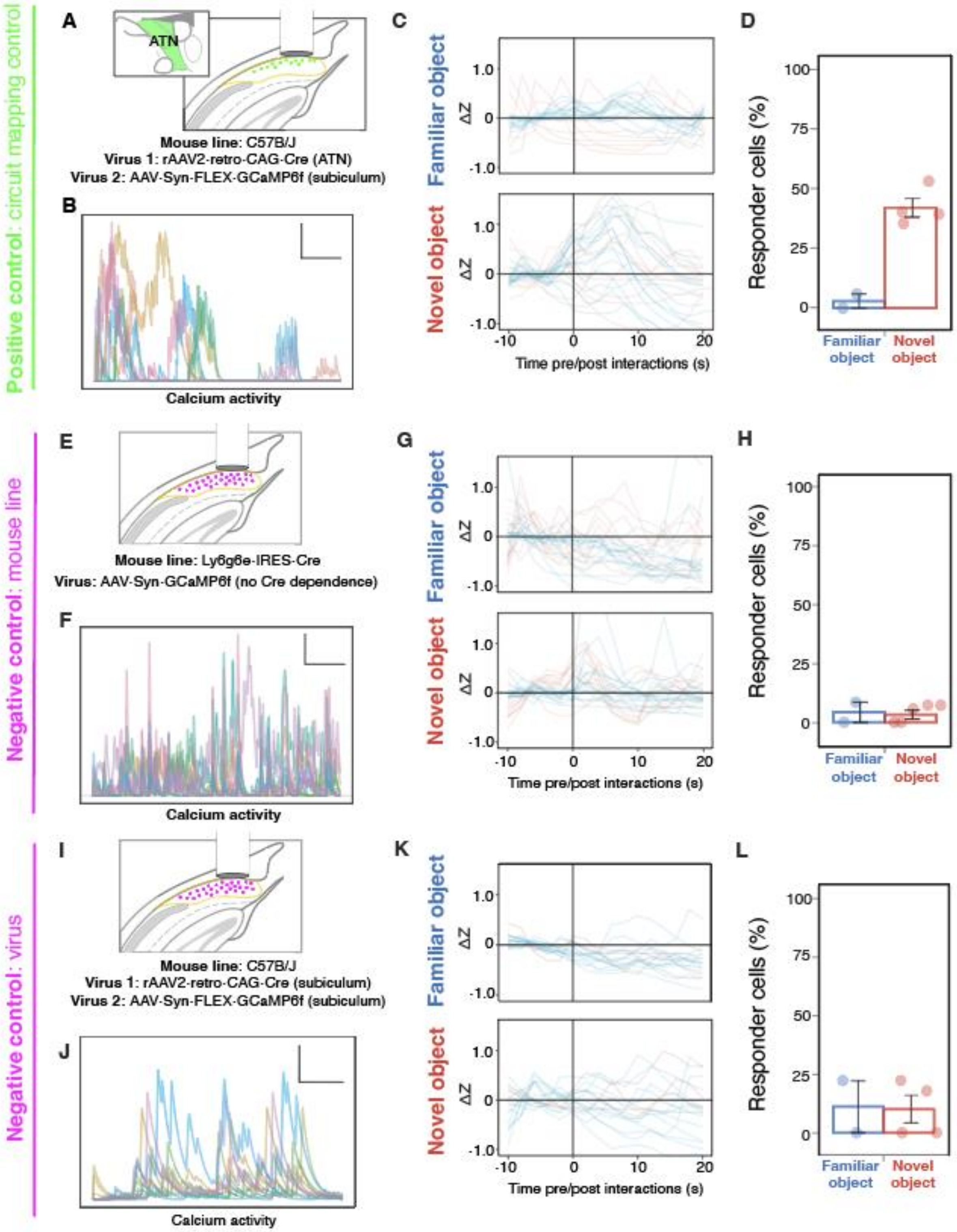

Supplemental Fig. 7. Controls for ovoid neuron calcium activity assessed *in vivo*.

(**A**) Schematic illustrating imaging of ovoid activity broadly across subiculum neurons, performed in a wild-type mouse, with retrograde virus strategy used to selectively drive GcaMP6f expression

in ATN-projecting subiculum neurons. (**B**) Calcium traces for neurons targeted in A. Different colors denote different cells. Scale bar: 10 seconds, with y-axis having arbitrary units. (**C**) Object-induced cellular activity in response to a familiar object (top) and a novel object (bottom) during the short-term retrieval session, for the strategy illustrated in A. Individual lines illustrate individual cells, with color denoting animal (n = 2 animals). (**D**) Percentage of neurons that respond to a familiar (blue) and novel (red) object, for the strategy illustrated in A (n=2 animals, with activity pooled across encoding and 1-day retrieval sessions. Data are presented as mean values +/- SEM. (**E-H**) As in A-D, but for imaging pyramidal cells broadly across subiculum neurons using a non-Cre-dependent GcaMP6f injected into the subiculum (n = 2 animals). (**I-L**) As in A-D, but for imaging pyramidal cells broadly across subiculum neurons using spatially co-injected rAAV2-retro-CAG-CRE and AAV-Syn-FLEX-GCaMP6f in the subiculum (n = 2 animals). Data are presented as mean values +/- SEM.

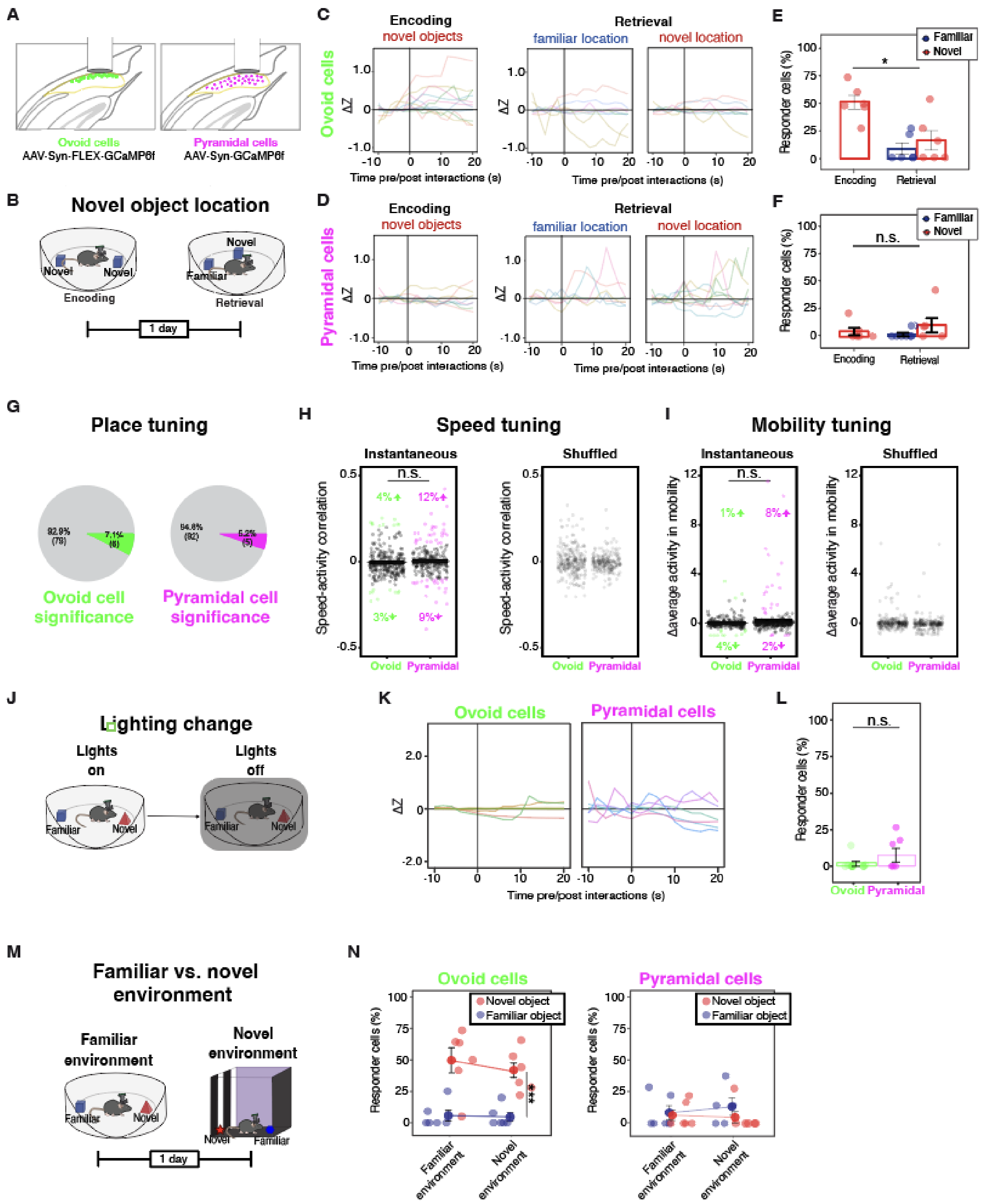

Supplemental Fig. 8. Ovoid cells lack responses to a variety of non-object forms of novelty.

(**A**) Viral and imaging strategy for assessing activity in ovoid neurons (left) and pyramidal neurons (right) with 1-photon calcium imaging, with approximate GRIN lens placement. (**B**) Schematic of novel object location behavioral assay. (**C**) Event-triggered averaging plots for ovoid cells, involving novel objects during encoding on the training session (left) and on retrieval during the test session for novel and familiar object locations (right). Different colors denote the mean cellular

activity of an individual animal. (**D**) As in C, but for pyramidal cells. (**E-F**) The percentage of cells that classify as a responder cell for novel objects during encoding (red, left), as well as familiar and novel object locations on retrieval (blue and red at right, respectively) for ovoid (**E**) (n = 6 encoding novel, n = 6 retrieval novel, n = 6 retrieval familiar; p = 0.035, Kruskal-Wallis test) and pyramidal cells (**F**) (n = 6 encoding novel, n = 6 retrieval novel, n = 6 retrieval familiar; p = 0.46, Kruskal-Wallis test). Data are presented as mean values +/- SEM. (**G**) Place tuning for ovoid (left) and pyramidal cells (right) from an open field recording with ovoid cells showing place cell tuning in 7.1% of cells and pyramidal cells showing place cell tuning in 5.2% of cells. (**H**) Left: speed scores of individual cells, across all recording sessions, for ovoid neurons (green) and pyramidal neurons (magenta). Values reflect instantaneous correlations between running speed and cellular activity, with colored points denoting cells that are significantly correlated with speed, with cellular percentages are provided. Right: as at left, but for shuffled analysis by staggering cellular activity 20 seconds relative to instantaneous locomotion (n = 236 pyramidal cells, n = 273 ovoid cells; pyramidal real vs shuffled: p = 0.47; ovoid real vs shuffled: p = 0.126; pyramidal real vs ovoid real: p = 0.08, two-sided Mann-Whitney U test). (**I**) Left: mean cellular activity for ovoid and pyramidal neurons during mobility, normalized to mean levels at immobility. Colored points denote cells that are significantly modulated by mobility, with cellular percentages are provided. Right: as at left, but for shuffled analysis by staggering cellular activity 20 seconds relative to mobility (n = 236 pyramidal cells, n = 273 ovoid cells; pyramidal real vs shuffled: p = 0.23; ovoid real vs shuffled: p = 0.96 ; pyramidal real vs ovoid real: p = 0.22, two-sided Mann-Whitney U test). (**J**) Schematic of light-changes to room. (**K**) As in C,D, but for summarizing responses aligned to timing of lighting change. (**L**) Percentage of cells designated as responders following lighting change (n = 6 pyramidal, n = 6 ovoid; p = 0.24, two-sided Mann-Whitney U test). Data are presented as mean values +/- SEM. (**M**) Schematic of changing general context from familiar environment to a novel environment. (**N**) Responder cells to familiar and novel objects, across familiar and novel environments, for both ovoid cells (left) and pyramidal cells (right) (n = 6 animals in all cases; ovoid x object: p = 2.3e-4; pyramidal x object: p = 0.37, Kruskal-Wallis test). Data are presented as mean values +/- SEM.

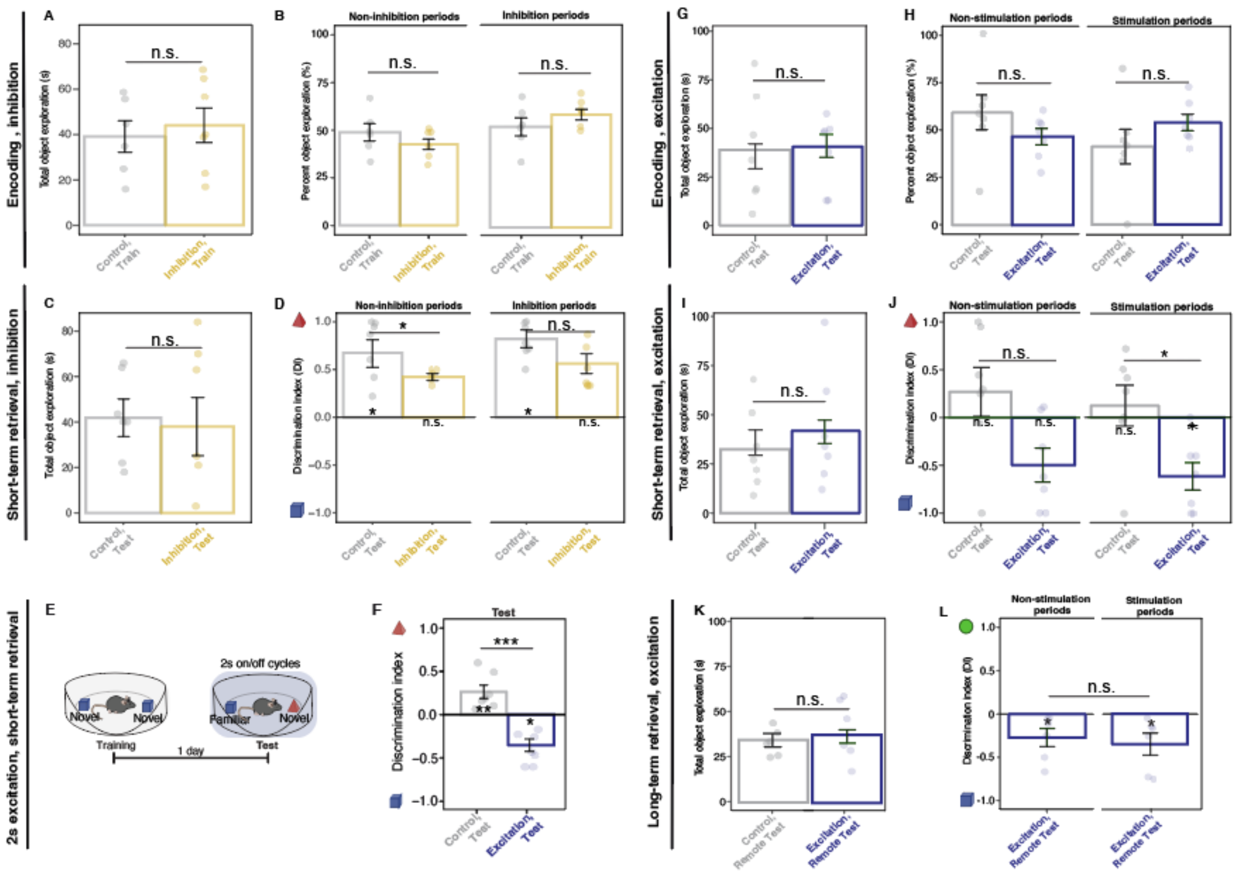

Supplemental Fig 9. Object exploration and discrimination indices in encoding, short-term retrieval, and long-term retrieval for inhibition and excitation.

(A) Total object exploration between control and ArchT-inhibited mice during entire encoding assay (n = 6 control, n = 7 ArchT; *p* = 0.57, two-sided Mann-Whitney U test). Data are presented as mean values +/- SEM. (**B**) Object exploration during inhibition vs. non-inhibition periods for data in A (non-inhibition *p* = 0.29, inhibition *p* = 0.29, two-sided Mann-Whitney U test). Data are presented as mean values +/- SEM. (**C**) As in A, but for ArchT-inhibited mice during the short-term retrieval session (n = 7 control, n = 6 ChETA; *p* = 0.67, Mann-Whitney U test). Data are presented as mean values +/- SEM. (**D**) Discrimination index for non-inhibition periods and inhibition periods on short-term retrieval day for data in B (non-inhibition *p* = 0.038, inhibition *p* = 0.12; inhibition from 0; control *p* = 0.035, ovoid *p* = 0.064, non-inhibition from 0; control *p* = 0.031, ovoid *p* = 0.13, two-sided Mann-Whitney U test). Data are presented as mean values +/- SEM. (**E**) Schematic for novel object recognition and optogenetic excitation of ovoid neurons with 2 second pulse durations during short-term retrieval. (**F**) Discrimination indices for control and ovoid mice on test day, for paradigm shown in E (n = 7 control, n = 7 ovoid; *p* = 3.1e-4, ovoid from 0 *p* = 7.8e-3, control from 0 *p* = 0.016, two-sided Mann-Whitney U test). Data are presented as mean values +/- SEM. (**G,H**) As in A,B, but for ChETA-excited mice during the (n = 7 control, n = 7 ChETA; total object exploration *p*= 0.82, non-stimulation *p* = 0.28, stimulation *p* = 0.28, two-sided Mann-Whitney U Test). Data are presented as mean values +/- SEM. (**I,J**) As in C,D, but for ChETA-excited mice (n = 7 controls, n = 7 ChETA; total object exploration: *p* = 0.52, non-

stimulation p = 0.054, stimulation p = 0.034, stimulation from 0: controls p = 0.53, ovoid p = 0.035, test stimulation from 0: controls p = 0.37, ovoid p = 0.075, two-sided Mann-Whitney U test). Data are presented as mean values +/- SEM. (**K**) As in G, but for long-term retrieval assay (n = 6 control, n = 6 ChETA; p = 0.59, two-sided Mann-Whitney U test). Data are presented as mean values +/- SEM. (**L**) Discrimination index for non-stimulation periods and stimulation periods on from long-term retrieval assay for ChETA-excited mice (n = 6; p = 0.48, stimulation p = 3.1e-2, non-stimulation p = 3.1e-2, two-sided Mann-Whitney U Test). Data are presented as mean values +/- SEM.
