# Supplementary material for: Atypical hippocampal excitatory neurons express and govern object memory
Source: Nat Commun. 2025 Feb 12;16:1195. doi: 10.1038/s41467-025-56260-8 (PMC11822006; doi:10.1038/s41467-025-56260-8)
Supplement: Supplementary file 2 — Description of Additional Supplementary Files [file 41467_2025_56260_MOESM2_ESM.pdf]

Description of Additional Supplementary Files

Movie S1. ATN-targeting subiculum neurons lack axon collaterals to other brain regions. Visualization of ATN-project subiculum neurons via brain clearing and light sheet imaging. For sparse labeling, neurons were labeled by rAAV2-retroEF1a-Flpo injected into the ATN and AAV8-hSynCon/Fon-EYFP injected into the dorsal subiculum.

Movie S2. Ovoid cells comprise a thin lamina and generally lack radial oblique dendrites. Visualization of ovoid neuron dendritic morphologies via brain clearing and light sheet imaging. For sparse labeling, neurons were labeled by rAAV2-retro-EF1a-Flpo injected into the ATN and AAV8-hSyn-Con/Fon-EYFP injected into the dorsal subiculum.
