# Supplementary material for: Atypical hippocampal excitatory neurons express and govern object memory
Source: Nat Commun. 2025 Feb 12;16:1195. doi: 10.1038/s41467-025-56260-8 (PMC11822006; doi:10.1038/s41467-025-56260-8)
Supplement: Supplementary file 5 — Reporting Summary [file 41467_2025_56260_MOESM5_ESM.pdf]

Corresponding author(s): Dr. Mark Cembrowski

Last updated by author(s): 12/10/2024

Reporting Summary

Nature Portfolio wishes to improve the reproducibility of the work that we publish. This form provides structure for consistency and transparency in reporting. For further information on Nature Portfolio policies, see our Editorial Policies and the Editorial Policy Checklist.

Statistics

For all statistical analyses, confirm that the following items are present in the figure legend, table legend, main text, or Methods section.

n/a	Confirmed	
☐	☒	The exact sample size (*n*) for each experimental group/condition, given as a discrete number and unit of measurement
☐	☒	A statement on whether measurements were taken from distinct samples or whether the same sample was measured repeatedly
☐	☒	The statistical test(s) used AND whether they are one- or two-sided<br>*Only common tests should be described solely by name; describe more complex techniques in the Methods section.*
☐	☒	A description of all covariates tested
☐	☒	A description of any assumptions or corrections, such as tests of normality and adjustment for multiple comparisons
☐	☒	A full description of the statistical parameters including central tendency (e.g. means) or other basic estimates (e.g. regression coefficient) AND variation (e.g. standard deviation) or associated estimates of uncertainty (e.g. confidence intervals)
☐	☒	For null hypothesis testing, the test statistic (e.g. F, t, r) with confidence intervals, effect sizes, degrees of freedom and P value noted<br>*Give P values as exact values whenever suitable.*
☒	☐	For Bayesian analysis, information on the choice of priors and Markov chain Monte Carlo settings
☒	☐	For hierarchical and complex designs, identification of the appropriate level for tests and full reporting of outcomes
☐	☒	Estimates of effect sizes (e.g. Cohen's *d*, Pearson's *r*), indicating how they were calculated

Our web collection on statistics for biologists contains articles on many of the points above.

Software and code

Policy information about availability of computer code

Data collection	Software used to collect imaging data was Mesoscale Imaging System (Translucence BioSystems), Arvivis Vision 4D (Carl Zeiss Microscopy, Germany), and Zen (Carl Zeiss Microscopy, Germany). Software used to collect electrophysiological data was PClamp11 software (Molecular Devices, Sunnyvale, CA, USA).
Data analysis	Code used to analyze scRNA-seq data was done with R (RRID:SCR_001905) using a combination of Seurat v4 (RRID:SCR_007322) and in-house code. Custom code used for multiplexed in situ hybridization analyses is available open source (hhttps://github.com/cembrowskilab/RUHi). Other publicly available code used in this manuscript includes the NEURON simulation environment (Hines and Carnevale, 1997), the UCLA Miniscope DAQ software (https://github.com/daharoni/Miniscope_DAQ_Software), the UCLA Wire-free Miniscope software (https://github.com/daharoni/wire-free-miniscope), and the Minian analysis pipeline (https://github.com/denisecailab/minian). Custom scripts in the R environment were used to analyze acquired data.

For manuscripts utilizing custom algorithms or software that are central to the research but not yet described in published literature, software must be made available to editors and reviewers. We strongly encourage code deposition in a community repository (e.g. GitHub). See the Nature Portfolio guidelines for submitting code & software for further information.

Data

Policy information about availability of data

All manuscripts must include a data availability statement. This statement should provide the following information, where applicable:

- Accession codes, unique identifiers, or web links for publicly available datasets
- A description of any restrictions on data availability
- For clinical datasets or third party data, please ensure that the statement adheres to our policy

Accession codes, unique identifiers, and web links are available for publicly available datasets as applicable. The data generated in this study are provided in the Supplementary Information/Source Data file.

Code from this manuscript are provided publicly through Figshare (https://figshare.com/projects/Atypical_hippocampal_excitatory_neurons_express_and_govern_object_memory/223434).

Research involving human participants, their data, or biological material

Policy information about studies with human participants or human data. See also policy information about sex, gender (identity/presentation), and sexual orientation and race, ethnicity and racism.

Reporting on sex and gender	NA
Reporting on race, ethnicity, or other socially relevant groupings	NA
Population characteristics	NA
Recruitment	NA
Ethics oversight	NA

Note that full information on the approval of the study protocol must also be provided in the manuscript.

Field-specific reporting

Please select the one below that is the best fit for your research. If you are not sure, read the appropriate sections before making your selection.

☒ Life sciences ☐ Behavioural & social sciences ☐ Ecological, evolutionary & environmental sciences

For a reference copy of the document with all sections, see nature.com/documents/nr-reporting-summary-flat.pdf

Life sciences study design

All studies must disclose on these points even when the disclosure is negative.

Sample size	No statistical methods were used to determine sample size. The sample sizes were determined by constraints of experiments and standards in the field.
Data exclusions	Non-neuronal cells and interneurons were removed from the scRNA-sequencing dataset. No other data were excluded.
Replication	Imaging, histology, behaviour and electrophysiology were replicated across multiple field-of-views (FOVs), multiple slices, animals, as applicable as stated in the manuscript.
Randomization	In behavioural data, objects and locations were counterbalanced. Experimental groups were generally conducted in parallel and under identical conditions. Animals were randomly assigned to groups.
Blinding	Researchers were blinded to genotype when scoring behavioural data, all other data collection was unblinded.

Reporting for specific materials, systems and methods

We require information from authors about some types of materials, experimental systems and methods used in many studies. Here, indicate whether each material, system or method listed is relevant to your study. If you are not sure if a list item applies to your research, read the appropriate section before selecting a response.

Materials & experimental systems

n/a	Involved in the study
☒	☐ Antibodies
☒	☐ Eukaryotic cell lines
☒	☐ Palaeontology and archaeology
☐	☒ Animals and other organisms
☒	☐ Clinical data
☒	☐ Dual use research of concern
☒	☐ Plants

Methods

n/a	Involved in the study
☒	☐ ChIP-seq
☒	☐ Flow cytometry
☒	☐ MRI-based neuroimaging

Animals and other research organisms

Policy information about studies involving animals; ARRIVE guidelines recommended for reporting animal research, and Sex and Gender in Research

Laboratory animals	Adult C57/B6 mice and Ly6g6e-IRES-cre mice were used for all experiments. Mice were maintained on a 12-h light-dark cycle with ad libitum access to food and water.
Wild animals	NA
Reporting on sex	Mice of both sexes were used.
Field-collected samples	NA
Ethics oversight	All experimental procedures involving mice were approved by the University of British Columbia AnimalAre Committee and/or Janelia Research Campus Institutional Animal Care and Use Committee.

Note that full information on the approval of the study protocol must also be provided in the manuscript.

Plants

Seed stocks	NA
Novel plant genotypes	NA
Authentication	NA
